# Intranasal Inoculation of Cationic Crosslinked Carbon Dots‐Adjuvanted Respiratory Syncytial Virus F Subunit Vaccine Elicits Mucosal and Systemic Humoral and Cellular Immunity

**DOI:** 10.1002/mco2.70146

**Published:** 2025-03-24

**Authors:** Hong Lei, Aqu Alu, Jingyun Yang, Cai He, Jie Shi, Weiqi Hong, Dandan Peng, Yu Zhang, Jian Liu, Furong Qin, Xiya Huang, Chunjun Ye, Lijiao Pei, Xuemei He, Hong Yan, Guangwen Lu, Xiangrong Song, Xiawei Wei, Yuquan Wei

**Affiliations:** ^1^ Laboratory of Aging Research and Cancer Drug Target State Key Laboratory of Biotherapy and Cancer Center National Clinical Research Center for Geriatrics West China Hospital Sichuan University Chengdu Sichuan People's Republic of China

**Keywords:** respiratory syncytial virus (RSV), intranasal vaccine, crosslinked carbon dot (CCD), adjuvant, mucosal immunity

## Abstract

Respiratory syncytial virus (RSV) causes severe acute lower respiratory tract infections, especially in infants and the elderly. Developing an RSV vaccine that promotes a robust mucosal immune response is necessary to successfully prevent viral transmission and the development of severe disease. We previously reported that crosslinked carbon dots (CCD) may be an excellent adjuvant candidate for intranasal (IN) protein subunit vaccines. Considering the strong immunogenicity of RSV prefused F protein (preF), we prepared an IN RSV vaccine composed of the CCD adjuvant and the preF protein as antigen (CCD/preF) and evaluated the induced antigen‐specific humoral and cellular immunity. We found that IN immunization with the CCD/preF vaccine elicited strong serum IgG responses and mucosal immunity, including secreted IgA antibodies, tissue‐resident memory T (T_RM_) cells, and antigen‐specific B cells, which lasted for at least 1 year. In addition, a combination of intramuscular and IN immunization with CCD/preF vaccine induced stronger systemic and mucosal immunity. Together, this study proved the high immunogenicity of the CCD/preF vaccines and supported the university of the mucosal CCD adjuvant, supporting further development of the CCD/preF vaccine in larger animal models and clinical studies.

## Graphical abstract

We prepared a CCD‐adjuvanted preF vaccine for intranasal delivery against RSV infection. Three‐dose intranasal administration of this vaccine induced long‐lasting humoral and cellular immunity against RSV F protein at both local sites and systemic immune organs. We then optimized the CCD/preF vaccine by combining IM and IN immunization, which induced stronger systemic and mucosal immunity.

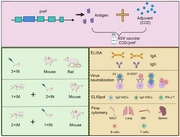

## 1 Introduction

Respiratory syncytial virus (RSV) is a ubiquitous seasonal human pneumoviruses that causes frequent upper and lower respiratory tract infections worldwide [1, 2]. It causes over 33 million infections and 300,000 hospitalizations globally every year, particularly among infants, young children, and the elderly [2, 3]. In the United States, 18% of elderly people hospitalized with RSV disease are admitted to the intensive care unit, and 26% die within 1 year after admission [4]. More importantly, the immune responses induced by RSV exposure can only protect against disease severity in the lower respiratory tract rather than infection, resulting in recurrent RSV infection in susceptible individuals [5, 6]. The high prevalence of RSV infection has caused serious health and economic burden to society, making the prevention of RSV infection a key work of the World Health Organization (WHO) [7]. In the 1960s, vaccination with the formalin‐inactivated RSV vaccine candidates led to enhanced respiratory disease after RSV exposure [8]. Later, the investigators found that the prefusion conformation of fusion glycoprotein (preF) is a highly immunogenic component of RSV, which accelerated the development of universal and safe preventive measures. Up to now, two humanized monoclonal antibodies (palivizumab and nirsevimab) and three vaccines (GSK's Arexvy, Pfizer's Abrysvo, and Moderna's mRESVIA) have been approved against RSV infection [9, 10, 11, 12, 13, 14]. The approved RSV vaccines are all administered via intramuscular (IM) injection, effectively reducing the risk of severe illness and mortality [15, 16]. However, they cannot induce effective mucosal immune responses as intranasal (IN) vaccines do [17]. The presence of prior mucosal secretory IgA is correlated closely with reduced RSV infection in infected volunteers [18]. IgA is a polymerized antibody located in both upper and lower respiratory tracts and provides sterilizing protection at the site of infection [19, 20, 21]. Previous studies indicated that intranasally administered vaccines can induce high levels of secretory IgA and establish tissue‐resident effector and memory T cells with the ability to rapidly perform innate and adaptive functions [22, 23, 24]. As yet, there are no IN vaccines approved against RSV.

Recombinant protein vaccines have been widely developed for mucosal delivery due to their super safety and ease of manufacture [25]. The fusion glycoprotein (F) on the surface of RSV usually exists as a trimer and plays a key role in the process of virus invasion [26]. During this process, the F protein undergoes substantial and irreversible conformational changes from the preF state before membrane fusion to a postfusion state (postF). PreF contains more immunogenic epitopes than postF, making the preF protein an attractive target in vaccine development [27, 28]. Administration of the stabilized preF induces higher levels of neutralizing antibodies against RSV than the postF protein [29]. However, owing to the low immunogenicity of pure proteins, adjuvants are usually required to stimulate host immunity and promote immune persistence by extending the residence time of vaccine antigens at the mucosal site [30, 31, 32]. Various mucosal adjuvants have been developed for IN vaccines, such as polymeric materials, agonists of pattern recognition receptors, cytokines, etc. [17]. Despite the great progress, no mucosal adjuvants have received official approval for human use for intranasal delivery due to toxicity concerns. Therefore, it is important to develop a safe and effective adjuvant to facilitate the development of intranasal vaccines.

In our previous study, we prepared a novel mucosal adjuvant named cationic cross‐linked carbon dots (CCDs), which showed excellent adjuvanticity for an intranasal protein subunit vaccine for the respiratory transmitted severe acute respiratory syndrome coronavirus 2 (SARS‐CoV‐2) [33]. Carbon dots (CDs) are spherical, nontoxic, and biocompatible nanoparticles that have been extensively explored in the delivery of genes, drugs, and antigens [34, 35, 36, 37]. To improve the encapsulating efficiency, we synthesized CCDs by connecting CDs with a linker via a ring‐opening reaction, thereby attracting protein antigens with anionic polypeptides to create nanoparticles based on electrostatic interactions. The CCD/RBD‐HR vaccine induced strong, broad, and long‐term protective immunity against viral infection after three‐dose intranasal immunizations [33]. Meanwhile, intranasal immunization with the CCD‐containing vaccine showed great tolerability in mice, without significant changes in the complete blood count, serum chemistry panel tests, and histological alterations in the vital organs [33]. The immunized mice were completely protected from SARS‐CoV‐2 infection, with no signs of exacerbated inflammation in the lungs, indicating a low incidence of vaccine‐enhanced respiratory disease [33].

In this study, we continued to investigate the efficacy of CCD‐adjuvanted intranasal RSV vaccines to explore the generalizability of the CCD adjuvant. We encapsulated the recombinant preF subunit protein with CCDs for intranasal delivery. Upon vaccination, the activation of humoral and cellular immunity was assessed in different animal models. Meanwhile, we explored the effect of various vaccination routes on the immune responses induced by the CCD/preF vaccine. We discovered significantly elevated levels of serum IgG antibodies specific to both preF and postF which lasted for at least 1 year after IN administration with CCD/preF. More importantly, substantial increases were observed in antigen‐specific mucosal IgA titers and the proportion of tissue‐resident memory and cytotoxic T cells in the lung, indicating the establishment of remarkably strong humoral and cellular immunity locally. Combining IN and IM immunization with CCD/preF shows advantages over pure IN immunization by inducing noninferior mucosal immunity but stronger systemic immune responses. This study evaluated the effectiveness of the intranasal CCD/preF vaccine candidate and explored the underlying mechanisms to deepen our understanding of the protective immunity against RSV.

## 2 Results

### 2.1 IN CCD/preF Vaccine Induced RSV‐Specific Mucosal IgA and Serum IgG Responses That Neutralized Virus Infection

**FIGURE 1:**
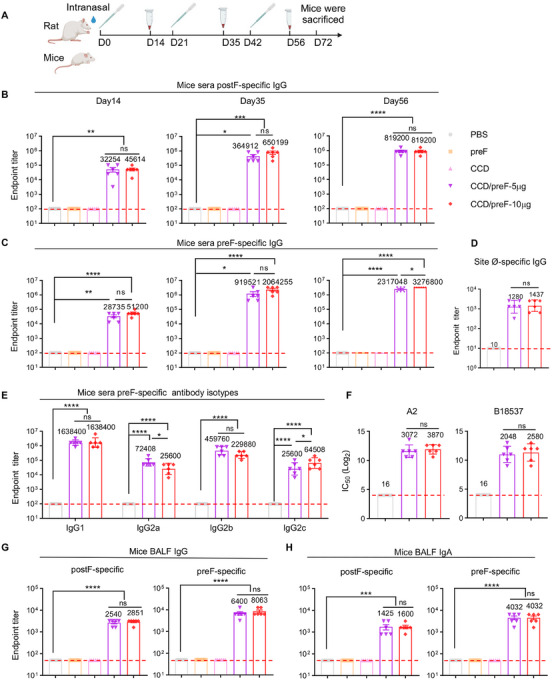
Intranasal immunization with the CCD/preF vaccine induced strong antibody responses. (A) A graphical illustration of the immunization schedule. Mice were immunized intranasally with CCD/preF vaccine on days 0, 21, and 42. On days 14, 35, and 56 after the first dose, postF‐ (B) and preF‐specific (C) IgG antibodies in the serum were evaluated with ELISA. (D) Site Ø‐specific IgG antibody titers were measured by D25‐competition ELISA. (E) preF‐specific IgG isotypes in the serum were assessed on day 56, including IgG1, IgG2a, IgG2b, and IgG2c. (F) Neutralization of the RSV A2 and B18537 strains’ infection into HEp‐2 cells by the immune sera on day 56. preF‐ and postF‐specific IgG (G) and IgA (H) titers in the BALF were determined with ELISA on day 72. *n* = 6. Data were displayed as mean ± SEM. P values were analyzed with One‐way ANOVA and Tukey's multiple comparison test. ****p < 0.0001, ***p < 0.001, **p < 0.01, and *p < 0.05. ns: not significant.

Based on our previous studies and dose optimization, we immunized mice with 50/5 µg of CCD/preF intranasally on days 0, 21, and 42 [33, 38]. To explore whether the addition of vaccine dose brings advantages over anti‐viral immunity, we vaccinated another group of mice with 100/10 µg of CCD/preF intranasally. Correspondingly, rats were immunized with 200/20 µg or 400/40 µg of CCD/preF via intranasal drops, respectively. Animals receiving PBS, naked preF, or the CCD adjuvant were defined as control groups. Serum from immunized animals was obtained 14, 35, and 56 days after the initial immunization, and bronchoalveolar lavage fluid (BALF) was obtained on day 72 to determine preF‐ and postF‐specific antibodies (Figure 1A). Enzyme‐linked immunosorbent assay (ELISA) analysis indicated that immunization with preF protein or CCD adjuvant cannot arouse postF‐ or preF‐specific antibodies in sera (Figure 1B,C). In contrast, antigen‐specific IgG antibodies in sera increased significantly on day 14 with titers of >10^4^ in mice (Figure 1B,C) and >10^2^–10^3^ in rats immunized with CCD/preF (Figure S1). After one boost, the sera IgG titers ascend to >10^5^ against postF and 10^6^ against preF in mice (Figure 1B,C), which reached >10^4^ specific to postF and 10^5^ against preF protein in rats (Figure S1A,B). The second booster of the intranasal CCD/preF further increased serum antibody titers, which reached over 10^7^ against preF in mice on day 56. No statistically significant differences were observed in the anti‐preF and anti‐postF serum IgG levels between low‐ and high‐dose groups of CCD/preF except for a slight enhancement of anti‐preF IgG antibodies in the high‐dose group on day 56, indicating that immunization with 5 µg of preF antigen is sufficient to induce substantial humoral immune responses in the presence of CCD adjuvant. Since the magnitudes of antibody response targeting site Ø is related to neutralizing capacity, we applied D25 competition ELISA to investigate if the tested serum antibodies effectually targeted this preF exclusive site. After the third dose, mice sera from the vaccine groups induced high anti‐site Ø IgG titers (Figure 1D).

Subtype analysis revealed that IgG1, IgG2a, IgG2b, and IgG2c antibodies against preF antigen were all enhanced in mice immunized with CCD/preF (Figure 1E). F‐specific antibodies can be used to predict virus‐neutralization antibodies against RSV [27, 39]. To determine the functions of antibodies, we infected HEp‐2 cells with the RSV A2 and B18537 strains and used the collected sera to neutralize viral infection at different dilutions. The results showed that both low and high‐dose CCD/preF vaccines induced high levels of neutralizing antibodies against RSV infection with titers of > 2000 (Figure 1F). These results indicated that CCD/preF can induce strong antigen‐specific humoral immune responses in the sera of intranasally vaccinated animals.

Next, we analyzed the production of antigen‐specific antibodies in BALF, including IgA and IgG. As expected, superior anti‐preF IgG and IgA responses were induced in mice inoculated with CCD/preF, but not in mice vaccinated with naked preF protein, CCD, or PBS (Figure 1G,H). Although slightly decreased compared with preF‐specific antibodies, IN CCD/preF administration also induced high levels of mucosal IgG and IgA antibodies specific to postF. These results supported that CCD is an excellent mucosal adjuvant for the recombinant subunit vaccine of RSV to induce strong mucosal and systemic antibody responses.

### 2.2 preF‐Specific IgG^+^ and IgA^+^ ASCs Induced in Local and System by IN CCD/preF Vaccine

**FIGURE 2:**
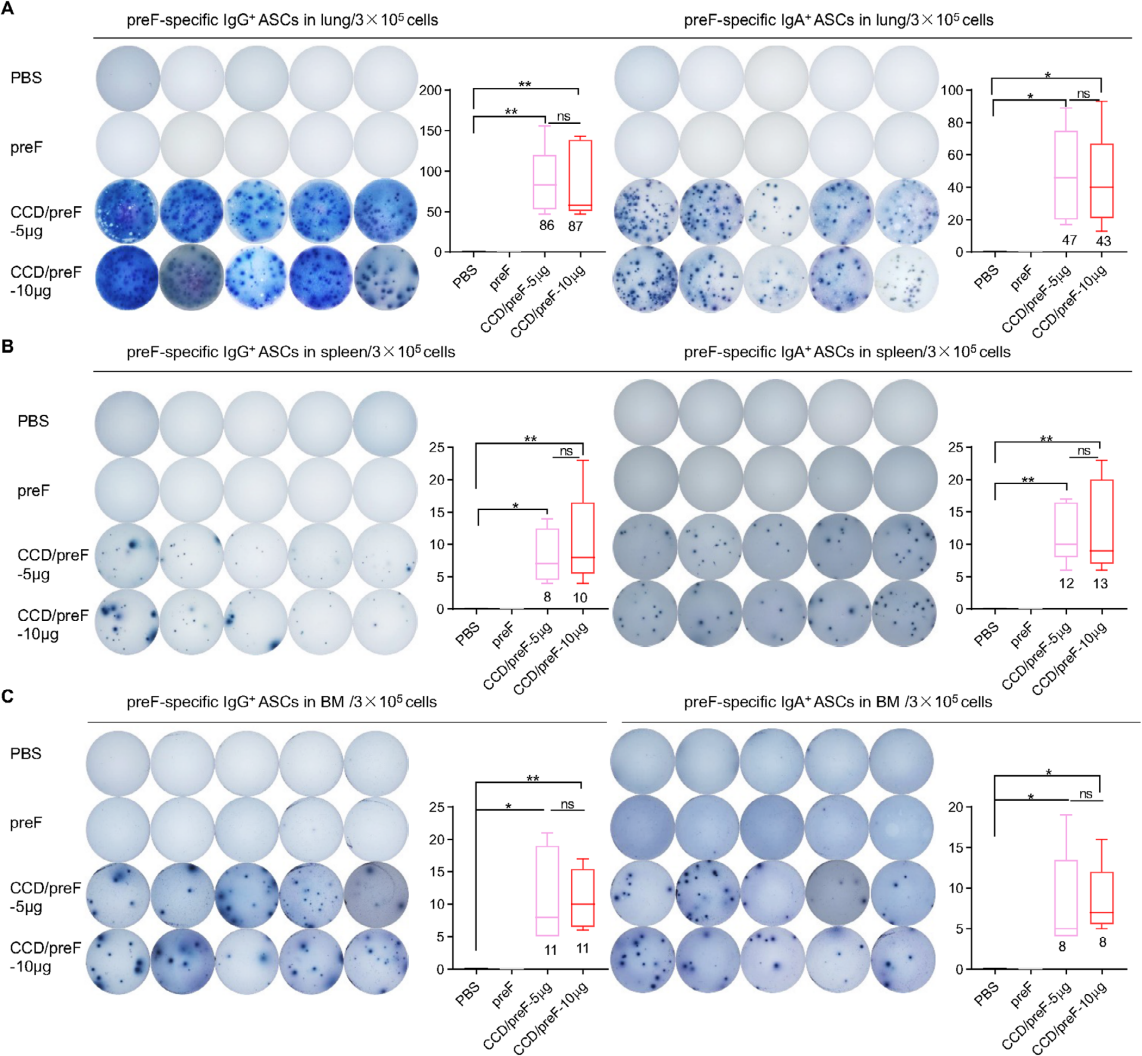
Intranasal immunization with the CCD/preF vaccine activated preF‐specific IgG^+^ and IgA^+^ ASCs. On day 72 after the prime, the immunized mice were sacrificed. Lymphocytes in the lungs (A), spleen (B), and BM (C) were isolated to determine preF‐specific IgG^+^ and IgA^+^ ASCs with ELISpot. Left: images of spot‐forming cells; Right: quantification of spot‐forming cells. *n* = 5. The middle line represents the median while the whisker shows the data range. P values were analyzed with One‐way ANOVA and Tukey's multiple comparison test. **p < 0.01 and *p < 0.05. ns: not significant.

Apart from humoral immunity, cellular immunity is also an essential part of antiviral immunity [40]. Antibody‐secreting cells (ASCs), derived from activated B cells, may differentiate into extracollicular cells or form germinal central responses within secondary lymphoid organs. ASCs are considered the mainstay of humoral immune responses because they can produce a large number of antibodies for host defense continuously [41, 42]. To study the underlying mechanisms, the immunized mice were sacrificed 1 month after the last immunization. Cells from the lungs, spleen, and bone marrow (BM) were isolated and added to preF‐coated wells for ASC detection. Enzyme‐linked immunospot assay (ELISpot) showed that IN immunization with low‐ and high‐dose CCD/preF induced comparable levels of antigen‐specific IgG^+^ and IgA^+^ ASCs in lung, BM, and spleen, but not in PBS and naked protein controls (Figure 2A–C). Meanwhile, preF‐specific IgG^+^ and IgA^+^ ASCs in the lung (Figure 2A) were superior to those in the spleen (Figure 2B) and BM (Figure 2C), indicating that the IN CCD/preF vaccine outperformed in activating antigen‐specific ASC responses producing mucosal antibodies.

### 2.3 Superior Antigen‐Specific B and T Cell Immunity Induced by IN CCD/preF Vaccine at Local Sites

**FIGURE 3:**
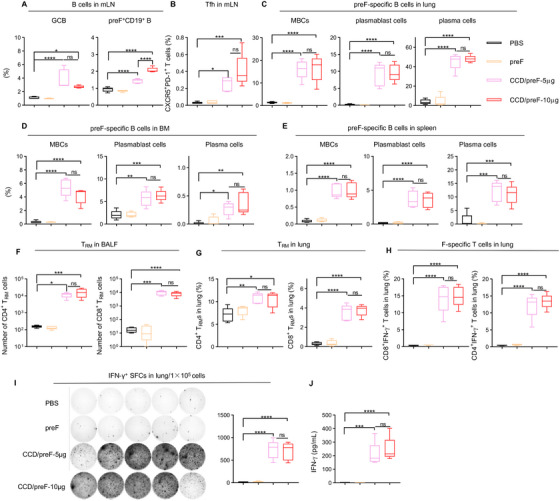
Intranasal immunization with the CCD/preF vaccine induced strong B and T cell immunity. Seventy‐two days after the prime, the immunized mice were sacrificed and BALF, mLN, lung tissues, BM, and spleen were collected. The frequencies of GCB (CD19^+^GL‐7^+^CD95^+^) and preF‐specific CD19^+^ B cells in the mLN (A), Tfh (CD4^+^CXCR5^+^PD‐1^+^) cells in the mLN (B), preF‐specific MB cells, plasmablast cells, and plasma cells in the lung (C), BM (D), and spleen (E) were assessed with flow cytometry (FCM). The numbers of CD4^+^ and CD8^+^ T_RMs_ in the BALF (F) and lung (G) were evaluated with FCM. (H) The frequency of CD4^+^ and CD8^+^ T cells producing IFN‐γ was assessed with FCM after intracellular cytokine staining. (I) Lymphocytes isolated from the lungs were re‐stimulated with F‐peptide pools for 24 h to determine preF‐specific lymphocytes that secret IFN‐γ with ELISpot. Left: images of spot‐forming cells; Right: quantification of spot‐forming cells. (J) After re‐stimulation, the IFN‐γ levels in the supernatants were analyzed with ELISA. *n* = 5. The middle line represents the median while the whisker shows the data range. P values were analyzed with One‐way ANOVA and Tukey's multiple comparison test. ****p < 0.0001, ***p < 0.001, **p < 0.01, and *p < 0.05. ns: not significant.

Activation of local and systemic B‐ and T‐cell responses after vaccination was analyzed by flow cytometry (FCM). Germinal centers play a key role in the proliferation of B cells and the generation of memory B cells (MB) [43], whereas follicular helper T cells (Tfh) are important in the regulation of high‐quality antibody response [32]. Thus, we detected GCB (CD19^+^GL‐7^+^CD95^+^) and Tfh (CD4^+^CXCR5^+^PD‐1^+^) cells in mediastinal lymph nodes (mLN) and found that the CCD‐adjuvanted RSV IN vaccine significantly increased the frequency of GCB (Figure 3A) and Tfh (Figure 3B) in mLN. preF protein is fluorescently labeled to probe preF‐specific B cells. preF‐specific B cell (preF^+^CD19^+^) elevation was observed in the mLN of the vaccine group compared with immunization with preF protein (Figure 3A). Plasmablasts induce an early burst of antigen‐specific antibodies after vaccination, whilst plasma cells can continuously secrete large numbers of antibodies with high affinity [44]. preF‐specific memory B cells (MBCs, CD138^−^CD19^+^CD38^+^), plasma cells (IgD^−^CD19^−^CD138^+^) and plasmablast cells (IgD^−^CD19^+^CD138^+^) in the lung (Figure 3C), BM (Figure 3D), and spleen (Figure 3E) were remarkably elevated in mice inoculated with CCD/preF vaccines regardless of the dosage, further demonstrating the activation of B cell immune responses.

Mucosal tissue‐resident memory T cells (T_RM_s) have been reported to have the potential for rapid host defense against viral infection in situ [45, 46]. Therefore, we evaluated the production of mucosal T_RM_s following IN administration of the CCD/preF vaccine. Mice that received the CCD/preF vaccine, but not pure preF protein, produced significant CD4^+^ and CD8^+^ T_RM_ cells in BALF (Figure 3F) and the lungs (Figure 3G). The lymphocytes obtained in lung tissues were stimulated by the F‐peptide pools. We then used intracellular cytokine staining (ICS) and ELISpot to assess the generation of interferon‐γ (IFN‐γ). The frequencies of IFN‐γ^+^CD4^+^ and IFN‐γ^+^CD8^+^ T cells (Figure 3H) and the total number of IFN‐γ secreting cells (Figure 3I) were remarkably increased in the lungs of mice vaccinated with CCD/preF. At the same time, ELISA results indicated that lung lymphocytes from the CCD/preF vaccinated mice secreted a large quantity of IFN‐γ cytokines into the supernatants after peptide restimulation (Figure 3J). Nevertheless, IN vaccination with CCD/preF failed to activate effective splenic T cells producing IFN‐γ against preF antigen (Figure S2). These data suggested that the IN CCD/preF vaccine can induce antigen‐specific T cell immune responses at the local sites instead of systemic immune organs.

### 2.4 IN CCD/preF Vaccine Elicits Long‐term Immune Responses for Over One Year

**FIGURE 4:**
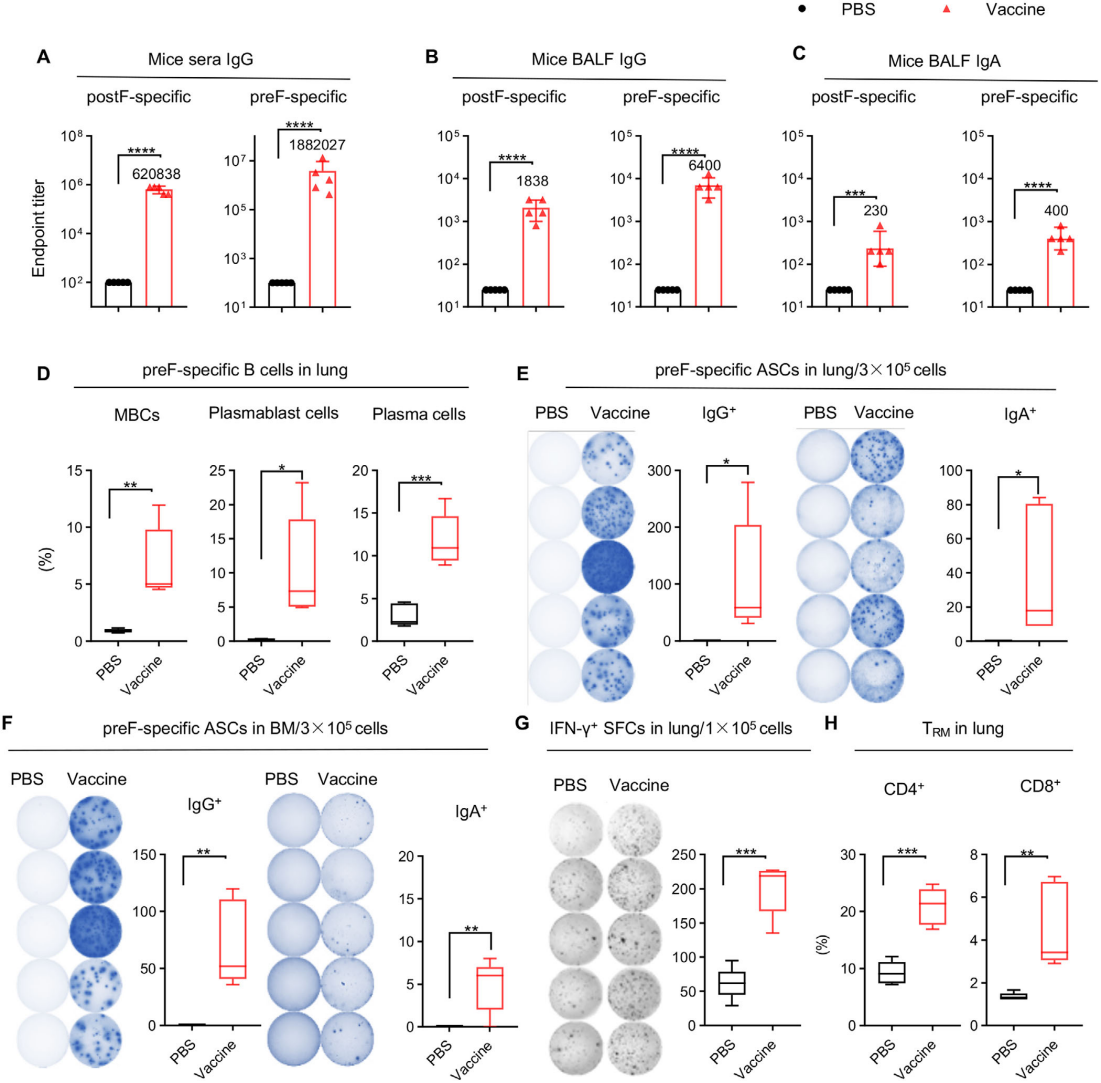
The long‐term immune responses induced by the intranasal CCD/preF vaccine. In 1 year after the first immunization, mice sera and BALF were collected to evaluate postF‐ and preF‐specific IgG (A, B) and IgA antibodies (C) with ELISA. In 1 year, mice were sacrificed to obtain lung and BM tissues. (D) The frequency of preF‐specific MB cells, plasmablast cells, and plasma cells in the lung were evaluated using FCM. Lymphocytes from the lung and BM were isolated and re‐stimulated with F‐peptide pools for 24 h to assess preF‐specific ASCs that produce IgG and IgA antibodies (E, F) or determine the number of preF‐specific SFCs producing IFN‐γ (G) with ELISpot. Left: images of spot‐forming cells; Right: quantification of spot‐forming cells. (H) The proportion of CD4^+^ and CD8^+^ T_RMs_ in the lung was evaluated with FCM. *n* = 5. Data were displayed as mean ± SD in (A–C). The middle line represents the median while the whisker shows the data range in (D–H). P values were conducted by Unpaired *t*‐tests. ****p < 0.0001, ***p < 0.001, **p < 0.01, and *p < 0.05.

The durability of vaccines’ efficacy is of great importance to avoid repeated vaccination. After immunization with CCD/preF (5 µg) vaccine, the preF‐ and postF‐specific IgG levels in sera did not decline to half of the day 56 levels by day 365, indicating an antibody half‐life of over 365 days (Figure 4A). The titers of antigen‐specific IgG in BALF showed no or only a slight decreasing trend on day 365 after immunization (Figure 4B). However, the titers of preF‐ and postF‐specific IgA in BALF reduced to less than 1/10 on day 365 compared with day 56 (Figure 4C). These results suggested that the IN CCD/preF vaccine can induce long‐lasting humoral immunity for up to 1 year.

We next assessed long‐term cellular immunity after IN immunization with the CCD/preF vaccine on day 365. A sharp increase in the frequency of preF‐specific MBCs, plasmablast cells, and plasma cells was observed in the lung (Figure 4D). ELISpot assay further supported the activation of B cells in the lung and BM, with elevated levels of ASCs secreting preF‐specific IgG and IgA in the vaccine group compared with PBS (Figure 4E,F). Cells isolated from lung tissues in the CCD/preF vaccine group maintained high levels of IFN‐γ production after restimulation with F‐peptide pools (Figure 4G). Similarly, we discovered that lung CD4^+^ and CD8^+^ T_RM_ responses remained relatively high in the CCD/preF vaccine group (Figure 4H). Thus, the IN CCD/preF vaccine can induce high and durable humoral and cellular immune responses both locally and systemically for over 1 year.

### 2.5 Combining IM and IN Immunization of CCD/preF Vaccine Elicits Stronger Mucosal and Systemic Antibody Responses

**FIGURE 5:**
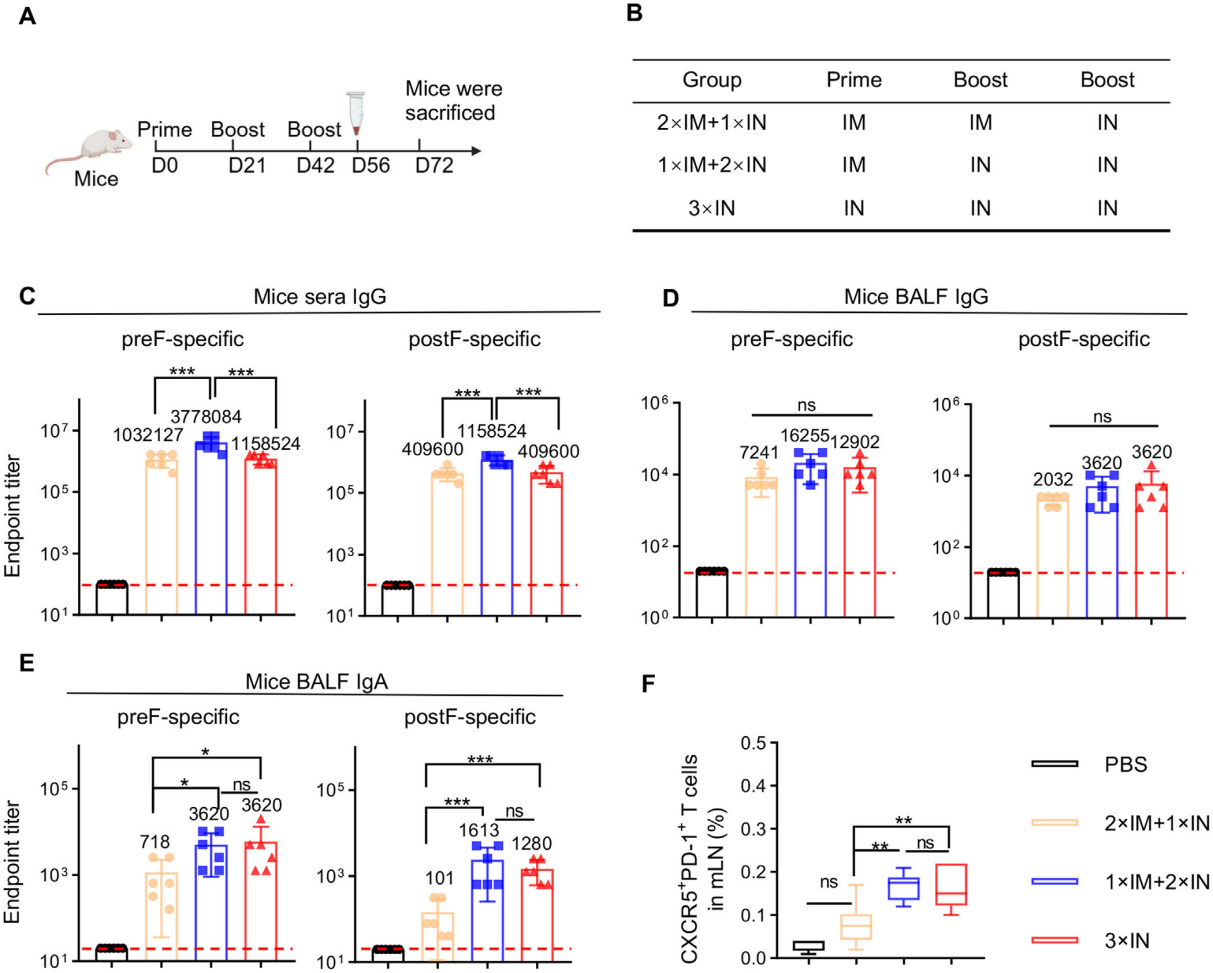
Combining IN and IM immunization with the CCD/preF vaccine enhanced humoral immunity. (A) A graphical illustration of the immunization schedule. (B) A description of the detailed immunization protocol. Mice were vaccinated on days 0, 21, and 42 through three doses of intranasal (3×IN) or heterologous immunization (2×IM+1×IN and 1×IM+2×IN). (C) On day 56, mice sera were collected to determine preF‐ and postF‐specific IgG titers with ELISA. (D‐F) On day 72, mice were sacrificed. BALF was collected to assess endpoint titers of preF‐ and postF‐specific IgG (D) and IgA (E) antibodies with ELISA. (F) The proportion of Tfh cells in the mLN was evaluated with FCM. *n* = 6. Data were displayed as mean ± SD in (C–E). The middle line represents the median while the whisker shows the data range in f. P values were analyzed with One‐way ANOVA and Tukey's multiple comparison test. ***p < 0.001, **p < 0.01, and *p < 0.05. ns: not significant.

We next investigated whether combining the conventional IM immunization could enhance the immunogenicity of the IN CCD/preF vaccine. Mice were immunized with different vaccination strategies (Figure 5A,B). NIH mice were intramuscularly primed with CCD/preF on day 0, followed by two‐dose boosters via IM and IN delivery (2×IM+1×IN) or only IN delivery (1×IM+2×IN) of the CCD/preF at intervals of 21 days. The mice receiving three IN doses of the CCD/preF vaccine (3×IN) or PBS were used as control (Figure 5B). On day 56, mice sera were collected for the detection of serum humoral immunity. On day 72, lungs, spleens, mLN, spleen, BALF, and BM were collected.

As shown in Figure 5C, the immunizations of 2×IM+1×IN and 3×IN elicited comparable titers of preF‐specific and postF‐specific IgG titers in serum, whereas 1×IM+2×IN immunization resulted in the highest IgG levels specific to both preF and postF. However, BALF preF‐ and postF‐specific IgG titers were not significantly boosted in the 2×IM+1×IN or 1×IM+2×IN groups, indicating that BALF IgG levels have reached a ceiling for the CCD/preF vaccine after IN administration (Figure 5D). Interestingly, 1×IM+2×IN immunization with CCD/preF vaccine induced comparably high BALF IgA titers compared with 3×IN immunization, whilst a modest reduction of preF‐specific and a sharp decrease of postF‐specific IgA titers were detected in the 2×IM+1×IN group (Figure 5E). Consistent with the results of antibody titers, 1×IM+2×IN and 3×IN immunization induced the highest percentage of Tfh cells in the mLN. In sharp contrast, 2×IM+1×IN vaccination showed limited activation of Tfh cells (Figure 5F). These results indicated that combining one IM prime and two IN boosters with CCD/preF could provoke comparable mucosal humoral immunity but superior serum IgG titers than pure IN delivery, whilst 2×IM+1×IN delivery compromised the antibody responses both locally and systemically.

### 2.6 Combining IM and IN Immunization of CCD/preF Vaccine Elicits Strong B and T Cell Immunity both Locally and Systemically

**FIGURE 6:**
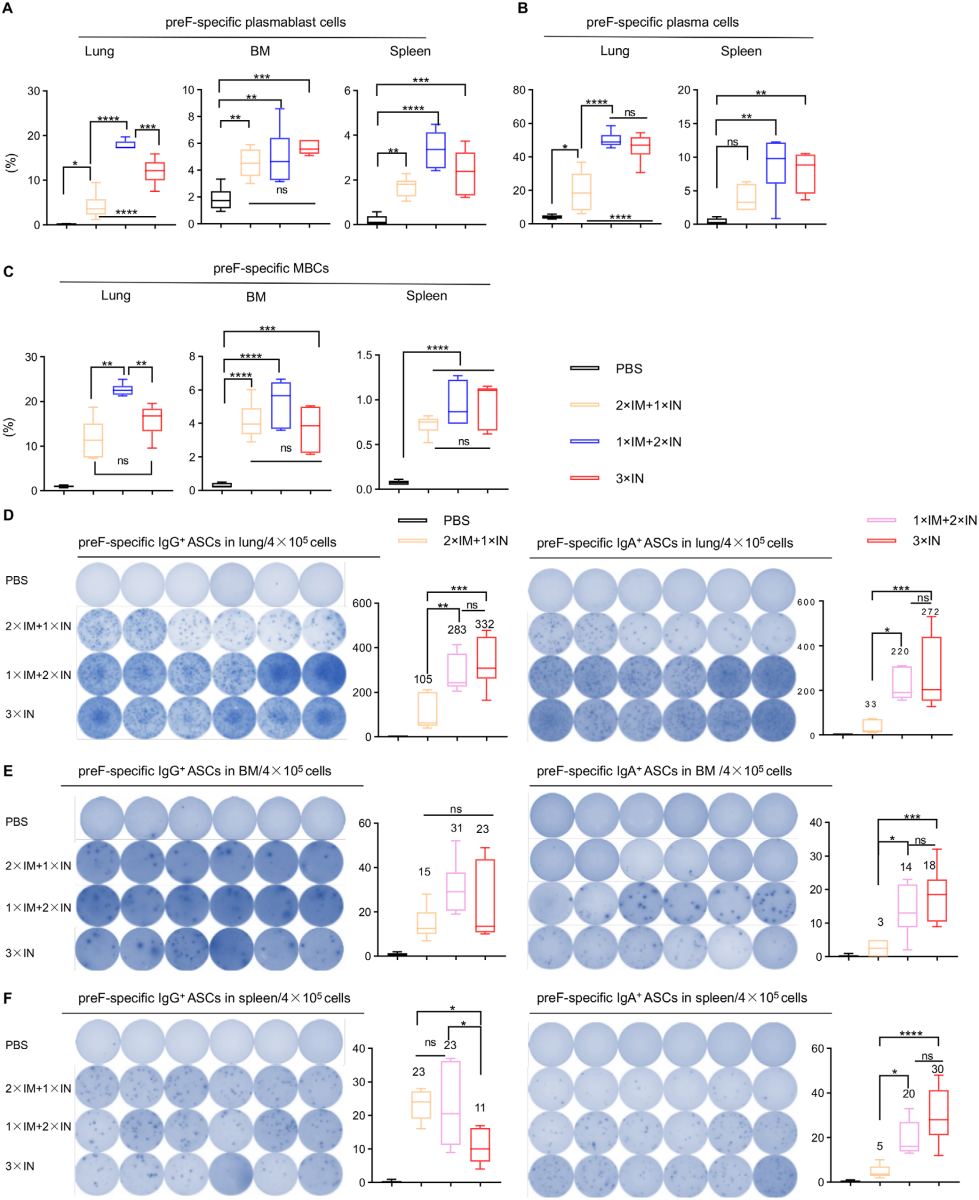
Combining IN and IM immunization with the CCD/preF vaccine enhanced B‐cell immunity. On day 72, mice were sacrificed to collect lung, BM, and spleen tissues. The proportion of preF‐specific plasmablast (A), plasma cells (B), and MB (C) cells in the lung, BM, and spleen were analyzed with FCM. Lymphocytes in the lungs (D), BM (E), and spleen (F) were isolated to determine preF‐specific IgG^+^ and IgA^+^ ASCs with ELISpot. Left: images of spot‐forming cells; Right: quantification of spot‐forming cells. *n* = 6. The middle line represents the median while the whisker shows the data range. *p*‐values were analyzed with one‐way ANOVA and Tukey's multiple comparison test. ****p < 0.0001, ***p < 0.001, **p < 0.01, and *p < 0.05. ns: not significant.

We measured antigen‐specific B cells in the lung, spleen, and BM with FCM on day 72 after the prime. We detected a significant increase of preF‐specific plasmablasts in the lung, BM, and spleen after administration of the CCD/preF vaccine, irrespective of vaccination route (Figure 6A). In the lung and spleen, 1×IM+2×IN immunization induced the highest proportion of antigen‐specific plasmablasts, followed by 3×IN immunization. We also discovered an escalation of preF‐specific plasma cells in the lung and spleen after vaccination with the CCD/preF vaccine, especially in the 1×IM+2×IN group (Figure 6B). These results further verified the noninferior capability of the 1×IM+2×IN vaccination strategy in inducing antigen‐specific antibodies than 2×IM+1×IN or 3×IN immunization. MB are long‐lived and can be activated and differentiate into ASCs rapidly after re‐exposure to the same antigen [47]. As expected, 1×IM+2×IN immunization induced a sharp increase in the frequency of MBCs in the lung, BM, and spleen. 2×IM+1×IN and 3×IN immunization also activated MBCs efficiently, but not as effectively as 1×IM+2×IN immunization (Figure 6C).

To evaluate the antigen‐specific B‐cell immunity, lymphocytes from the lung, BM, and spleen were isolated and subjected to an ELISpot assay with preF protein. As shown in Figure 6D–F, the CCD/preF vaccine induced preF‐specific ASCs producing IgG antibodies in the lung, BM, and spleen, regardless of different immunization routes. 2×IM+1×IN immunization is weaker in inducing IgG ASCs in the lung whereas 3×IN immunization is weaker in activating IgG ASCs in the spleen, making 1×IM+2×IN immunization the most stable and effective method to induce preF‐specific IgG‐producing B cell responses both locally and systemically. We also assessed the production of IgA‐producing ASCs after combining IM and IN immunization. We found that 1×IM+2×IN and 3×IN immunization induced comparatively higher levels of ASCs producing preF‐specific IgA antibodies in the lung, BM, and spleen compared with 2×IM+1×IN immunization. These results indicated that the combination of one IM prime and two IN booster immunizations can induce significantly higher B cell immune responses both at local sites and systemically.

**FIGURE 7:**
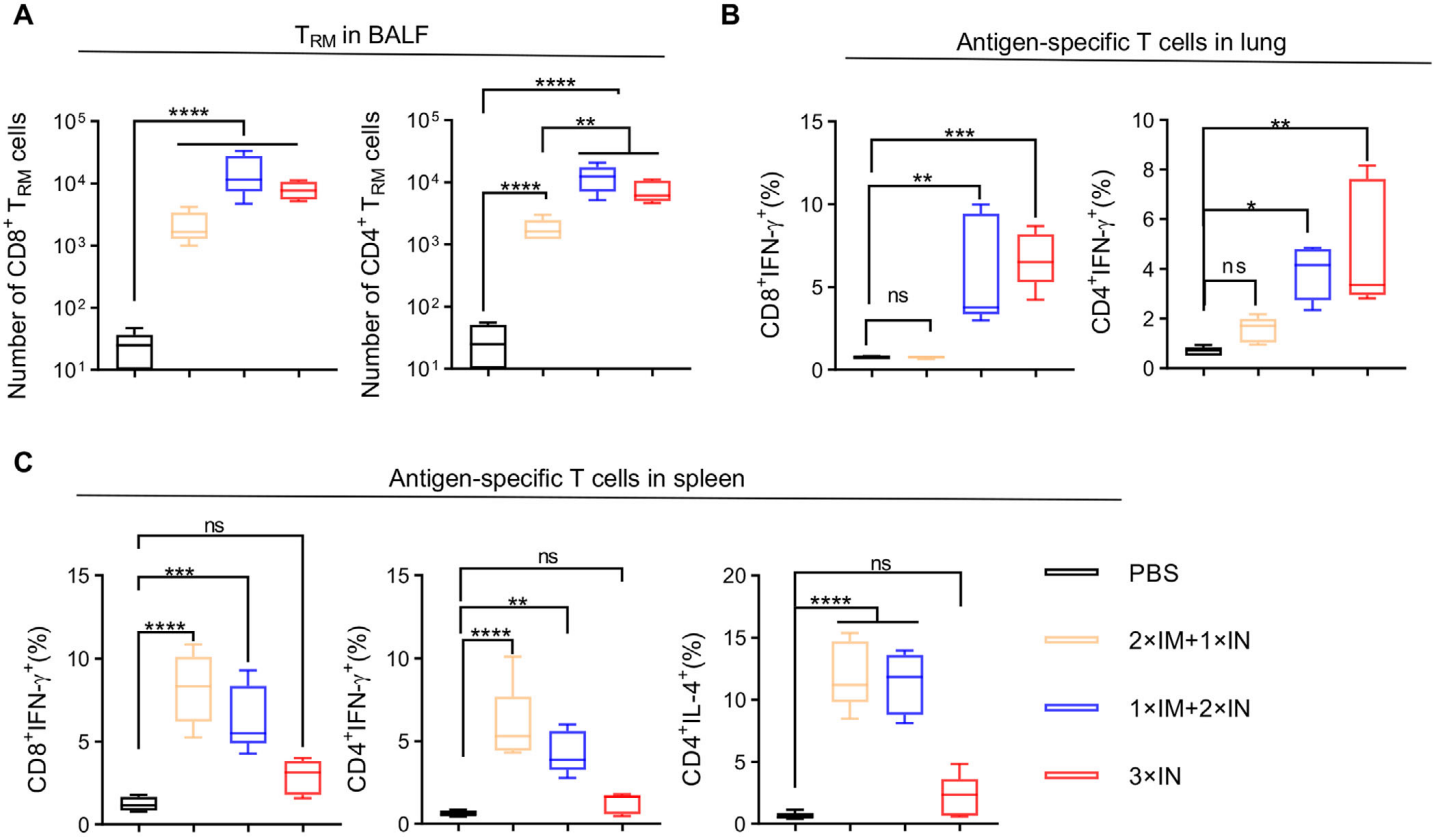
Combining IN and IM immunization with the CCD/preF vaccine enhanced T cell immunity. On day 72, mice were sacrificed to collect BALF, lung, and spleen. (A) The numbers of CD4^+^ and CD8^+^ T_RMs_ in the BALF were evaluated with FCM. Lymphocytes from lung (B) and spleen (C) were isolated and re‐stimulated with F‐peptide pools for 24 h to detect preF‐specific CD4^+^ and CD8^+^ T cells that secret IFN‐γ or IL‐4 cytokines with FCM. *n* = 5. The middle line represents the median while the whisker shows the data range. *p*‐values were conducted by one‐way ANOVA analysis followed by Tukey's multiple comparison test. ****p < 0.0001, ***p < 0.001, **p < 0.01, and *p < 0.05. ns: not significant.

We next analyzed whether the combination of IM and IN immunization of CCD/preF vaccine can induce superior T cell responses. In BALF, we found that 1×IM+2×IN immunization induced comparable numbers of CD8^+^ and CD4^+^ T_RM_ cells as 3×IN immunizations, in contrast to the decreased tendency of 2×IM+1×IN immunization (Figure 7A). Similar results were observed in the frequency of preF‐specific T cells in the lung. T cells in the lung tissues of the vaccinated mice were harvested and restimulated with F‐peptide pools before being analyzed with ICS. We found that both 1×IM+2×IN and 3×IN immunization induced high levels of CD8^+^IFN‐γ^+^ and CD4^+^IFN‐γ^+^ T cells in the lung, whereas 2×IM+1×IN immunization did not (Figure 7B). We found that 3×IN immunization with the CCD/preF vaccine is insufficient to elicit T‐cell responses in the spleen (Figure S2). However, combining one or two IM immunizations can overcome this obstacle by increasing the frequencies of preF‐specific CD4^+^ and CD8^+^ T cells producing IFN‐γ and/or IL‐4 cytokines in the spleen (Figure 7C). Therefore, combining IM and IN immunization can induce comparably strong antigen‐specific local immunity compared with IN immunization while maintaining the potential of CCD/preF in activating strong systemic immune responses.

## 3 Discussion

The global burden of disease caused by RSV has received increasing attention, especially in infants, older adults, and immunocompromised individuals. Great efforts have been made in developing RSV vaccines, leading to the approval of three vaccines within the past 2 years, including two vaccines based on recombinant preF protein (GSK's Arexvy and Pfizer's Abrysvo) and one mRNA‐based vaccine targeting preF (Moderna's mRESVIA) [12]. These vaccines are administered by IM injection, aiming to reduce disease severity in the lower respiratory tracts (LRTs) of RSV‐infected individuals. There is an urgent need for an RSV vaccine that could directly prevent RSV infection in the LRTs or even in the upper respiratory tracts, promoting the development of IN RSV vaccines. Several IN RSV vaccines have launched into clinical stages, mostly belonging to live‐attenuated vaccines and viral vectored vaccines [48, 49, 50]. However, their development was limited by the concerns of enhanced RSV diseases with inactivated RSV vaccine and genetic instability and antivector antibodies with viral vectored RSV vaccines. Subunit preF vaccines have shown excellent safety and immunogenicity and have taken a large market share via IM injection, inspiring us to explore IN RSV vaccines based on subunit proteins. Chiu's team developed a mucosal RSV F protein vaccine adjuvanted by an immunostimulatory bacterium‐like particle, which evoked systemic plasmablast responses and strong serum antibodies in healthy, seropositive adults [51]. However, their vaccine showed a dissatisfactory effect in inducing F protein site ∅‐specific antibodies and nasal IgA responses. By modifying the vaccine formulation with our self‐developed CCD adjuvant [33], our CCD/preF vaccine induced significant and long‐lasting site ∅‐specific IgG antibodies and mucosal IgA responses after IN immunization with either low or high doses (Figures 1D,H and 4C). Meanwhile, the preF antigens used in this study were designed based on the A2 strain of RSV but the induced antibodies effectively neutralized the infection of authentic A2 and B18537 strains in vitro (Figure 1F), suggesting its potential to elicit cross‐protective immunity against both strains.

Generally, the antibodies induced by natural RSV infection are short‐lived, causing repeated infection [18]. Here we show that IN delivery of CCD/preF established a robust humoral immunity and immune memory for no less than 1 year (Figure 4), which is consistent with our previous study [33] and was superior to other IN vaccine candidates concerning the durability of the immune response [51, 52]. Although serum antibodies, nasal IgA, and cellular immunity are all essential for protecting against symptomatic RSV infection, the dimeric mucosal secretory IgA (sIgA) is thought to be the most essential for preventing RSV infection at the site of infection [53, 54]. It was reported that higher levels of RSV‐specific nasal IgA were related to reduced RSV infection‐associated clinical symptoms whilst lower nasal IgA levels resulted in increased risks of RSV infection [18, 55]. Therefore, developing an RSV vaccine that could produce RSV‐specific nasal IgA is critical to prevent RSV infection. In our study, IN vaccination with the CCD/preF vaccine induced high levels of preF‐ and postF‐specific IgA antibodies in the BALF (Figure 1H), indicating its potential to inhibit the invasion of RSV at the mucosal sites.

Cellular immunity plays a vital role in viral clearance. IN CCD/preF vaccines activated a range of immune responses, from enhanced cytotoxic T cells in the lungs to T_RM_ responses in the lungs and BALF (Figure 3F–J). The establishment of T_RMs_ in the lungs and airways promoted more rapid control of RSV in the lungs after reinfection [56, 57]. Though different IN RSV vaccines have been developed, few have evaluated the activation of T_RM_ responses, especially in the airway [58, 59]. In our study, we found that IN immunization with the CCD/preF vaccine activated high levels of CD4^+^ and CD8^+^ T_RM_ cell responses in the lung and BALF, which remained strongly activated in 1 year after the prime immunization (Figure 3F,G and 4H). Upon antigen re‐exposure, local T_RM_ cells can rapidly produce inflammatory cytokines and differentiate into effector T cells to promote tissue antiviral resistance [17].

The currently licensed RSV vaccines are all administrated via IM injection, which induces remarkable levels of serum IgG and systemic cellular immunity, thus alleviating disease severity caused by RSV infection [60, 61]. Nevertheless, their capability in preventing RSV infection and nasal shedding is limited owing to the poor activation of mucosal immunity. It is believed that IN vaccines play safeguard roles at the viral entry sites by inducing mucosal immunity IgA and T_RMs_ [62]. Consistently, IN inoculation of the CCD/preF vaccines induced high levels of mucosal sIgA and T_RM_ responses as mentioned before. Meanwhile, magnitude titers of anti‐RSV serum IgG antibodies were induced by the IN CCD/preF vaccine, indicating the activation of both local and systemic immunity (Figure 1B–E; Figure S1). However, we found that IN immunization with CCD/preF showed limited efficacy in activating systemic cellular immunity (Figure S2), which is essential for viral clearance. Previous studies indicated that a combination of IM and IN vaccination may help overcome this obstacle [24, 63]. Inspired by this, we investigated whether a combination of IN and IM immunization can optimize the immunogenicity of the CCD/preF vaccine. By comparing different combination strategies, we discovered that IM priming followed by two IN booster vaccinations with the CCD/preF vaccine induced the strongest RSV‐specific systemic and mucosal immunity (Figure 5, 6, 7).

Despite these promising findings, this study is limited by the fact that we did not evaluate the protective effect of the CCD/preF vaccine against authentic RSV infection in animal models, also making it difficult to evaluate the risk of the major challenge in RSV‐vaccine development: vaccine‐enhanced respiratory disease (VERD) [64]. VERD was first discovered in children intramuscularly immunized with the formalin‐inactivated‐RSV vaccine, who experienced enhanced disease and lung inflammation [65, 66]. However, IN vaccination with live‐attenuated, viral vector‐based, or recombinant protein vaccines has not raised VERD concerns in either preclinical or clinical studies, highlighting the potential advantages of IN immunization as a safer option for RSV vaccine development. Meanwhile, considering the outstanding safety profile of recombinant protein vaccines [25] and the tested safety of our newly developed CCD adjuvant [33], it can be foreseen that the CCD/preF IN vaccine will have a low probability of causing VERD. Our future studies will focus on investigating the efficacy and safety of the IN CCD/preF vaccine in preclinical animal models including nonhuman primates to promote its clinical translation. The currently approved RSV vaccines are primarily for the elderly over 60 years old with a high risk of RSV‐infection‐induced LRT diseases [12]. Recently, the age limit of Arexvy has been expanded for high‐risk adults aged 50 to 59 years whereas Abrysvo has also been approved for adults aged 18 to 59 years old. The RSV vaccine market for children younger than 18 years of age, especially infants, is still a blank. The target population of our vaccine is the susceptible population for RSV, especially young children. The needle‐free route can overcome children's fear of IM injections and make immunization easier and more convenient because people can administer vaccines themselves.

## 4 Materials and Methods

### 4.1 Materials

preF (cat: 11049‐VNAS), Biotinylated‐preF (cat: 11049‐VNAS‐B), and postF (cat: 11049‐V08B) were obtained from SinoBiological. Horseradish peroxidase (HRP)‐conjugated anti‐mouse IgG (cat: 0107‐05) and IgA (cat: 1040‐05) antibodies and Horseradish peroxidase (HRP)‐conjugated goat anti‐rat IgG (Cat: 3030‐05) were provided by southern biotech. RSV A2 (cat: VR‐1540) and B18537 (cat: VR‐1580) were obtained from an American‐type culture collection (ATCC). D25 antibody (cat: PABL‐322) was purchased from Creative Biolabs.

### 4.2 CCD Preparation

The CCDs were synthesized according to our previous work [33]. Briefly, pre‐prepared CD‐1.8k and linkers were dissolved in ethanol followed by a reaction at 80°C for three days with N_2_ protection. Ethanol was adequately removed from the reaction mixture and mixed solvent (TFA and CH_2_Cl_2_) was added and stirred for 16 h at room temperature (RT). The solvent was then fully removed using vacuum evaporation. Dissolve the residue in deionized water and purify it using dialysis with 0.1N HCl solution (MWCO 3500) for 48 h and deionized water for 24 h. The final product was freeze‐dried to obtain CCD.

### 4.3 Vaccine Formulations and Vaccination of Animals

For vaccine preparation, 100 µg CCD was incubated with 5/10 µg of preF protein for 30 min at RT. Then, the volume was refilled to 50 µL with PBS. Six‐ to eight‐week‐old female NIH mice (SPF) were purchased from Beijing Vital River Laboratory Animal Technologies Co., Ltd (China) and raised in the animal facility (SPF) of the Animal Center of the State Key Laboratory of Biotherapy.

To identify the immune response induced by IN immunization, the mice were randomly divided into five groups: mice in the vaccine group were immunized intranasally with 50/5 µg or 100/10 µg doses of vaccine (CCD/preF, 50 µL/one mouse). Mice receiving 100 µg of CCD or 5 µg of the naked protein were used as the control. In the PBS group, mice were inoculated with an equal volume of PBS.

To investigate the efficacy of integrating IM and IN immunization, NIH mice (5 mice/group) were immunized with 5 µg preF antigen plus CCD adjuvant. One group of mice was inoculated with two doses of vaccine via the IM pathway and then received the third dose of vaccine via the IN pathway (2×IM+1×IN). Mice in the 1×IM+2×IN group were first immunized intramuscularly and then boosted twice via the IN route.

The 6‐week‐old rats (female) were immunized intranasally with three doses of 20 µg vaccine (200 µL per rat). The mice and rats were injected on days 0, 21, and 42. Sera were collected on days 14, 35, and 56, and BALF was collected on day 72 to assess the binding and neutralizing antibodies. All animal experiments in this study were approved by the Institutional Animal Care and Use Committee of Sichuan University.

### 4.4 Enzyme‐Linked Immunosorbent Assay

Anti‐preF and postF specific IgG and IgA in sera and BALF were identified by ELISA. First, the 96‐well plates (NUNC‐MaxiSorp; Thermo Fisher Scientific) were coated with preF and postF proteins (1 µg/mL) in the carbonate coating buffer and incubated overnight at 4°C. after the plates were washed three times by PBST (PBS with 0.1% Tween 20), 100 µL blocking buffer (PBST containing 1% BSA) was added and plates were incubated at a 37°C incubator for 1 h. After one washing with PBST, the double gradient diluted serum or BALF was added to the well and reacted for 2 h at 37°C. Then, the plates were washed three times and secondary antibodies (HRP‐conjugated anti‐mouse IgG, IgA, or anti‐rat IgG, 1:10,000, Abcam) were transferred to wells. After reacting for 1 h at 37°C, the plates were added with 100 µL/well of TMB (3,3′,5,5′‐tetramethyl biphenyl diamine) and developed fully at RT for 10 min. The reactions were quenched with 1 M H_2_SO_4._ In the end, the absorbance was detected at 450 nm on a microplate reader (Spectramax ABS; Molecular Devices).

To evaluate the concentration of IFN‐γ in the supernatant of lymphocytes, a mouse IFN‐γ ELISA Kit (CAT: 88‐8314‐88, Invitrogen) was used and we completed the whole experiment strictly in accordance with the instructions in the manual.

### 4.5 Competition ELISA Assay

A competition ELISA was done to measure the amounts of antibodies targeting the PreF exclusive antigenic site Ø in mice sera. Firstly, a SuperBiotin Quick labeling kit (Frdbio, China) was used to conjugate Biotin to the D25 antibody (referred to as a tracer). 96‐well ELISA plates were coated with 100 µL/well of PreF (2 µg/mL) and incubated overnight at 4°C. Then, the plates were washed with PBST and then blocked with a blocking buffer for 90 min at RT. The immune serum samples were serially diluted in a blocking buffer and then mixed in equal volumes with a tracer (8 ng/mL). After one washing of the plates, the tracer‐sample mixtures were added. Following three times wash, HRP‐labeled streptavidin (Cat: A0305, 1:5000, 100 µL/well, Beyotime, China) was added to the plates and incubated for 1 h at RT. Then, plates were washed three times and incubated with 100 µL/well of TMB substrate for 10 min at RT for full reaction. 100 µL/well of 1 M H_2_SO_4_ was added to stop the reaction. The optical density was measured at 450/620 nm with a microplate reader (Spectramax ABS; Molecular Devices). The serum dilution ratio at 50% inhibition of tracer binding to preF is the antibody titer.

### 4.6 Enzyme‐Linked Immunospot (ELISpot) Assay

The immunized mice were sacrificed on day 72 or 1 year after the prime immunization. preF‐specific IgG and IgA antibody‐secreting cells (ASCs) in the lung, bone marrow, or spleen were identified using ELISpot. 96‐well filtration plates (REF: MSIPS4W10, Millipore) were treated with 35% ethanol for 1 min and then washed five times with sterile water. Each well was added with 100 µL preF (3 µg/mL) and incubated overnight at 4°C. Following that, the plates underwent 5‐time washes with PBS. Subsequently, plates were blocked by RPMI‐1640 complete medium for at least 1 hat 37°C. Lymphocytes of the immunized mice were obtained with mouse lymphocyte isolation solution and added to the wells. The plates were kept in the incubator overnight and not moved during the process. After three washes with PBS, anti‐mouse IgG or IgA Ab (100 µL/well) was introduced to the wells. After being reacted at RT for 2 h, the plates underwent three rounds of washing with PBS and then added to TMB ELISpot substrate solution. After the formation of clear spots, the reaction was stopped by flowing water. Finally, the spates were naturally dried and the spots were counted on the S6 Ultra M2 ELISpot reader (CTL, USA).

To detect IFN‐γ‐secreting cells in the lungs of immunized mice, a mouse IFN‐γ ELISpot kit (CAT:3321‐4APT‐2) was used. Coated plates were washed with PBS, blocked by 1640 complete medium for 1 h, and then added with cells taken from the lung (1×10^6^/well). Cells in the wells were stimulated with F peptide pools for 16 h at 37°C and then washed with a PBS rinse. Plates were added with diluted detection antibody (100 µL/well) and incubated for 2 h at RT After washing with PBS, each well was added with 100 µL diluted streptavidin‐ALP and incubated for 1 h. Following that, plates were washed and BCIP/NBT‐plus were transferred into wells for spot formation.

### 4.7 Virus Neutralization Assay

Virus neutralization assay (VNA) was conducted using collected sera from vaccinated NIH mice. Briefly, sera were heat‐inactivated at 56°C for 30 min and then twofold serially diluted ranging from 16 to 32768. Then, the diluted sera were mixed with A2 or B18537 RSV viruses (2 ×10^4^ TCID50/mL) in equal volume. After incubation for 2 h at 37°C, 100 µL mixture was introduced into 96‐well plates containing HEp‐2 cells (5 ×10^4^/well, ATCC, CCL‐23). The HEp‐2 cells were incubated at 37°C for 1 week. The presence of cytopathogenic effects (CPE) was observed using a microscope. The lowest dilution resulting in 80% CPE inhibition was determined as the neutralizing antibody titer for the mice serum sample.

### 4.8 Flow Cytometry

The tissues were obtained on day 30 after the last vaccination. Mediastinal lymph nodes (mLN) were milled, incubated with red blood lysis solution, and filtered to get single cells. Lung tissues isolated from immunized mice were cut into small pieces and digested in digestive buffer (DMEM with type‐1 and type‐IV collagenases) in advance before treatment like mLN. Lymphocytes in the spleen can be obtained according to the instructions of the lymphocyte separation kit (DAKEWE, CAT: 7211011). Cells in all tissues were analyzed by a NovoCyte Flow Cytometer with NovoExpress 1.4.1 (ACEA Biosciences, Inc.).

Cells in mLN were stained by anti‐mouse antibodies including PerCP/Cyanine5.5‐CD3 (BioLegend; cat#100218), FITC‐CD4 (BioLegend; cat#130308), BV605‐CD19 (BioLegend; cat#11540), PE‐CD279 (PD‐1) (BioLegend; cat#135205), and BV421‐CD185 (CXCR5) (BioLegend; cat#145512) at 4°C for half an hour to determine follicular helper T cells (Tfh). To detect germinal center B cells (GCB) and preF‐specific B cells in mLN, cells were first treated with the Biotinylated‐preF for 30 min at RT. Following that, cells were washed once by PBS and then stained by PerCP/Cyanine5.5‐CD3, BV605‐CD19, PE‐Streptavidin (BioLegend; cat#405204) and BV421‐GL‐7 (BioLegend; Cat#144614).

To detect T_RM_s in BALF or lungs, the following antibodies were used: PerCP/Cyanine5.5‐CD3, FITC‐CD4, BV421‐CD8a (BioLegend; cat#100725), PE‐CD69 (BioLegend; cat#164204), PE/Cyanine7‐CD44 (BioLegend; cat#103030), and APC‐CD103 (BioLegend; cat#110906).

preF‐specific plasma cells, plasmablast cells, and memory B cells in the lung, bone marrow, or spleen can be analyzed simultaneously. After being treated with the Biotinylated‐preF, cells were stained by BV421‐CD4 (BioLegend; cat#100438), PerCP/Cyanine5.5‐CD19 (BioLegend; cat#152405), FITC‐IgD (BioLegend; cat#405704), APC‐GL‐7 (BioLegend; cat#144618), BV650‐CD138 (BioLegend; cat#142518), PE/Cyanine7‐CD38 (BioLegend; cat#165617) and PE‐Streptavidin. The immune cells in this study are gated as previously reported [33].

### 4.9 Statistical Analyses

All statistical analyses were performed using the GraphPad Prism 8.0 program. For comparisons between multiple groups, *p*‐values were calculated using unpaired student's *t*‐tests or one‐way ANOVAs followed by Tukey's multiple comparisons and expressed by notation. ****p < 0.0001, ***p < 0.001, **p < 0.01 and *p < 0.05. ns: not significant.

## Author Contributions

Yuquan Wei and Xiawei Wei conceived and supervised the research, and designed the experiments. Xiya Huang, Cai He, Hong Lei, Chunjun Ye, Dandan Peng, and Jie Shi performed vaccine formulation and vaccinations in mice, and binding antibodies assay. Hong Yan and Hong Lei performed a virus neutralization assay. Weiqi Hong, Yu Zhang, Jian Liu, and Furong Qin performed the ELISpot experiment. Xuemei He and Lijiao Pei performed flow cytometry. The CCD adjuvant was prepared by Xiangrong Song. Yuquan Wei, Xiawei Wei, Guangwen Lu, and Jingyun Yang analyzed and interpreted the data and assisted with the adjustments of directions and interpretation of the mechanistic aspects of the results. Aqu Alu and Hong Lei wrote the manuscript. All authors have read and approved the article.

## Ethics Statement

All animal experiments in this study were approved by the Institutional Animal Care and Use Committee of Sichuan University (ethical approval number: 20230227017).

## Conflicts of Interest

Yuquan Wei is an editorial board member of MedComm, but he has not been involved in the process of manuscript handling. The remaining authors declare no conflicts of interest.

## Supporting information



Supporting Information

## Data Availability

The data in this study are available from the corresponding author upon reasonable request.
