# Supplementary material for: Intranasal Inoculation of Cationic Crosslinked Carbon Dots‐Adjuvanted Respiratory Syncytial Virus F Subunit Vaccine Elicits Mucosal and Systemic Humoral and Cellular Immunity
Source: MedComm (2020). 2025 Mar 24;6(4):e70146. doi: 10.1002/mco2.70146 (PMC11933438; doi:10.1002/mco2.70146)
Supplement: Supplementary file 1 — Supporting Information [file MCO2-6-e70146-s001.docx]

Supplementary Information for

# Intranasal inoculation of cationic crosslinked carbon dots-adjuvanted respiratory syncytial virus F subunit vaccine elicits mucosal and systemic humoral and cellular immunity

Correspondence author. [yuquanwei@scu.edu.cn](mailto:yuquanwei@scu.edu.cn) (Editorial corresponding author), Xiawei Wei ([xiaweiwei@scu.edu.cn](mailto:xiaweiwei@scu.edu.cn)), Xiangrong Song ([songxr@scu.edu.cn](mailto:songxr@scu.edu.cn)), and Guangwen Lu (lugw@scu.edu.cn).

**Includes:**

Supplemental Figure 1-2

**Supplemental Figures**

SUPPLEMENTARY FIGURE 1 Intranasal immunization with the CCD/preF vaccine induced strong antibody responses in rats. Rats were immunized intranasally with CCD/preF vaccine on days 0, 21, and 42. On days 14, 35, and 56 after the first dose, postF- (A) and preF-specific (B) IgG antibodies in the serum were evaluated with ELISA. n=3. Data were displayed as mean ± SEM. P values were conducted by One-way ANOVA analysis followed by Tukey’s multiple comparison test. ****p < 0.0001, ***p < 0.001 and **p < 0.01. ns: not significant.

SUPPLEMENTARY FIGURE 2 Intranasal immunization with the CCD/preF vaccine did not activate T cell immunity in the spleen. On day 72 after the prime, the immunized mice (n=5) were sacrificed to evaluate preF-specific IFN-γ-producing T cells in the spleen with ELISpot after re-stimulation of F-peptide pools for 24h. The middle line indicates the median and the whisker shows the data range. ns: not significant.
